# Genetic and clinical determinants of tacrolimus accumulation in early liver transplantation: evidence for a combined genotype-antibiotic effect

**DOI:** 10.1080/07853890.2026.2643032

**Published:** 2026-03-12

**Authors:** Jinlin Guo, Shiying Feng, Zifan Feng, Jinyan Yang, Xinfeng Cai, Xingang Li, Rui Zhang

**Affiliations:** a Department of Pharmacy, Shanxi Provincial People’s Hospital, Taiyuan, China; b Department of Hepatobiliary Surgery, Liver Transplantation Center, First Hospital of Shanxi Medical University, Taiyuan, China; c Department of Pharmacy, Jincheng Second People’s Hospital, Jincheng, China; d Department of Pharmacy, Shanxi Province Cancer Hospital/Shanxi Hospital Affiliated to Cancer Hospital, Chinese Academy of Medical Sciences/Cancer Hospital Affiliated to Shanxi Medical University, Taiyuan, China; e Department of Pharmacy, Beijing Friendship Hospital, Capital Medical University, Beijing, China

**Keywords:** Tacrolimus, pharmacokinetics, liver transplant, genotype-antibiotic, cefoperazone, ABCB1

## Abstract

**Background:**

Tacrolimus is a first-line immunosuppressant essential for preventing graft rejection after liver transplantation, but its narrow therapeutic index and extensive interindividual pharmacokinetic variability complicate optimal dosing during the early postoperative phase.

**Methods:**

The pharmacokinetics of tacrolimus were analyzed in liver transplant recipients from longitudinal trough concentrations collected within the first 30 days of administration using non-compartmental models. Associations between pharmacokinetic indices and covariates were assessed using Spearman’s correlation, t-tests, ANOVA, Wilcoxon rank-sum, or Kruskal-Wallis tests, and genotype-drug combined effects by ANOVA. Protein models were generated by ColabFold and docked with drugs using CB-Dock2. Machine-learning models predicted pharmacokinetic parameters.

**Results:**

Tacrolimus trough accumulation dynamics showed interindividual variability, with the time to maximum trough concentration (t(C0,max)) ranging across patients. The ABCB1 rs2032582 AC genotype in recipients was associated with earlier t(C0,max) and greater accumulation, while donor CYP3A4 rs4646437 was linked to differences in a trough accumulation index. Cefoperazone/sulbactam administration was associated with more pronounced trough accumulation and shorter t(C0,max), and a combined genotype-antibiotic effect was observed, whereby rs2032582 AC carriers receiving cefoperazone/sulbactam reached peak trough levels earlier. Docking analyses suggested that cefoperazone may competitively bind ABCB1 at sites overlapping tacrolimus binding, particularly in the rs2032582 AC variant. Multiple biomarkers for liver function, coagulation, and hematologic indices were associated with pharmacokinetic metrics, findings supported by machine-learning models.

**Conclusions:**

These findings highlight a combined effect of genetic background and antibiotic use on tacrolimus trough exposure dynamics, providing mechanistic insight that may inform individualized monitoring and dosing in the early phase after liver transplantation.

## Introduction

Tacrolimus is a cornerstone immunosuppressant in solid-organ transplantation and is widely used in liver transplantation to prevent graft rejection [1]. Globally, its adoption has improved both short- and long-term outcomes for liver transplant recipients by reducing acute rejection *via* T-cell suppression [2]. Given the drug’s narrow therapeutic index and its critical role in graft survival and patient morbidity, detailed study of its pharmacokinetics (PK) in the liver-transplant setting is of great significance [3].

Drug transport processes have been increasingly recognized as important determinants of tacrolimus exposure and kinetics. The ATP-binding cassette transporter P-glycoprotein (P-gp; encoded by ABCB1) plays a key role in efflux of tacrolimus in intestinal enterocytes and hepatocytes, thereby limiting net absorption and promoting biliary excretion of unchanged drug or metabolites [4]. Clinical and mechanistic studies have shown that ABCB1 expression, polymorphisms, and function correlate with tacrolimus disposition and intracellular accumulation in transplant recipients, although results remain somewhat inconsistent [5]. Thus, transporters, e.g. P-gp (and potentially others), act in concert with metabolic enzymes to modulate the PK of tacrolimus.

In parallel with drug transport, recent population PK studies in liver transplant patients have examined the influence of genetic polymorphisms, e.g. CYP3A5 and CYP3A4, on drug clearance and dose requirements [6,7]. It is now well established that tacrolimus is primarily metabolized in the liver and intestinal wall by the CYP3A family of enzymes with the formation of multiple demethylated and hydroxylated metabolites (e.g. 13-O-desmethyl tacrolimus) [8]. These metabolic pathways contribute substantially to the pharmacokinetic variability observed in liver transplant patients.

Drug-drug interactions can alter exposure of tacrolimus and clinical outcomes. Concomitant CYP3A/P-gp inhibitors such as macrolide antibiotics (e.g. clarithromycin) and azole antifungals (e.g. posaconazole) have been shown to significantly increase tacrolimus concentrations by inhibiting both CYP3A4 and P-gp, necessitating dose adjustment and close monitoring to avoid toxicity [9–11]. In contrast, strong inducers like rifampin and anticonvulsants (e.g. phenytoin and carbamazepine) accelerate tacrolimus metabolism, often resulting in subtherapeutic levels and increased rejection risk [12,13]. Common antihypertensive agents, including calcium channel blockers like nifedipine, may also inhibit CYP3A activity and elevate tacrolimus levels [14]. These interactions underscore the importance of reviewing concomitant medications to optimize tacrolimus therapy in polypharmacy settings.

In clinical practice for liver transplantation, tacrolimus is often initiated early, typically on the day of surgery or within the first few hours post-transplant to achieve prompt immunosuppression [2]. However, the early post‐transplant period, typically the first few days to weeks after surgery, represents a unique and high-risk phase for tacrolimus therapy due to dynamic changes in graft function, absorption, metabolism and distribution, all of which contribute to markedly elevated pharmacokinetic variability [15]. Several studies have demonstrated that during this phase, tacrolimus bioavailability, clearance and volume of distribution fluctuate widely [16,17]. Despite a growing body of pharmacokinetic research, most studies have focused on stable or later-phase recipients rather than this highly dynamic early period, and comprehensive evaluation of key covariates, for example genetic polymorphisms, hematocrit or albumin levels, and concomitant medications, remains relatively uncommon [18–20]. Consequently, the ability to reliably individualize tacrolimus dosing during the early post-transplant phase remains limited, highlighting the clinical importance of characterizing early exposure patterns to inform dose adjustment in real-world settings.

Although intensive pharmacokinetic sampling is the gold standard for characterizing full concentration-time profiles, it is rarely feasible in immediate post-transplant patients because of surgical recovery, clinical burden, and ethical constraints [19]. Consequently, therapeutic drug monitoring (TDM) in routine clinical practice relies almost exclusively on longitudinal trough concentrations, which are used to guide dose adjustment and are known to correlate moderately with overall tacrolimus exposure at steady state [20,21]. Importantly, during the early non-steady-state phase, tacrolimus trough concentrations have been associated with biomarkers for inflammation, hepatic function and hematologic changes [22,23]. Therefore, analyzing the time-course of trough concentrations provides a pragmatic approach to investigate inter-patient variability in tacrolimus accumulation and the timing of steady-state attainment under real-world dosing conditions [21,24]. However, previous studies have highlighted that trough-only monitoring has limitations in predicting full pharmacokinetic profiles and peak exposure [21]. Thus, trough-based surrogate metrics, for example the maximum observed trough concentration, time to reach this maximum, and accumulation ratios, serve as descriptive indicators of accumulation behavior rather than classical single-dose pharmacokinetic parameters, allowing clinically meaningful comparisons while acknowledging methodological constraints [2,21,25].

In this study, PK of tacrolimus based on longitudinal trough concentration data during the first month after liver transplantation was systematically characterized using an integrated clinical and computational approach. Genetic (recipient and donor SNPs focusing on ABCB1 and CYP3A variants, respectively) and clinical factors were analyzed to identify determinants of PK variability. Structural docking analyses were conducted to explore molecular interactions among ABCB1 polymorphisms, tacrolimus, and concomitant medications. Finally, machine-learning models incorporating clinical, biochemical, and genetic predictors were developed to explain interindividual PK variability and identify key determinants governing tacrolimus disposition.

## Materials and methods

### Recruitment of participants

Patients were enrolled at Shanxi Provincial People’s Hospital and First Hospital of Shanxi Medical University between November 2024 and September 2025. The study was approved by the Ethics Committee of Shanxi Provincial People’s Hospital (No. 2024510) and Ethics Committee of First Hospital of Shanxi Medical University (KYLL-2024-281), and all participants or their legal guardians provided written informed consent before participation. Etiology of liver disease and baseline MELD score of participants are shown in Supplementary Dataset 1.

### Inclusion and exclusion criteria

Patients aged over 18 years who underwent liver transplantation and received a tacrolimus-based immunosuppressive regimen were included in the study. Exclusion criteria included the following: (1) severe postoperative complications that interfered with recovery of gastrointestinal function or prevented regular oral administration and scheduled tacrolimus trough monitoring. These conditions comprised persistent ileus or bowel obstruction, recurrent vomiting or gastric retention requiring prolonged gastrointestinal decompression, the need for long-term parenteral nutrition, severe intra-abdominal infection or sepsis with hemodynamic instability, and reoperation or interventional procedures that markedly compromised perioperative stability; (2) failure of liver function to improve after transplantation; (3) poor medication adherence; and (4) refusal to provide informed consent.

### Monitoring of tacrolimus

Tacrolimus was administered within 6 h after surgery and pre-dose (trough) concentrations were detected. During the first week after transplantation, drug concentrations were measured daily; during the second week, every other day; and thereafter, once every 3–4 days. Whole-blood tacrolimus concentrations were measured by chemiluminescent microparticle immunoassay using the Abbott ARCHITECT^®^ system. The assay’s lower limit of quantification was 0.5 ng/mL. Intraday and interday precision, expressed as coefficients of variation, were ≤4% and ≤5%, respectively. Calibration spanned 1–30 ng/mL, and quality control samples at 3.0, 12.0, and 25 ng/mL were included in each analytical run. To avoid confounding from strong drug-drug interactions, tacrolimus concentrations obtained after initiation of antifungal therapy were excluded from the pharmacokinetic analysis. During the analysis window, apart from the standard perioperative immunosuppressive regimen, no strong CYP3A or P-gp inhibitors or inducers (e.g. rifampin, phenytoin/carbamazepine and clarithromycin) and no antihypertensive medications were co-administered.

### Clinical and biochemical data collection

A comprehensive set of clinical and biochemical variables was collected by the Electronic Health Record of Shanxi Provincial People’s Hospital and First Hospital of Shanxi Medical University, including age, sex, height, weight, and BMI, as well as laboratory parameters reflecting hepatic function (alanine aminotransferase [ALT], aspartate aminotransferase [AST], total bilirubin, and albumin), renal function (serum creatinine), hematologic and coagulation indices (hematocrit, platelet count, prothrombin time [PT], activated partial thromboplastin time [APTT], thrombin time [TT], international normalized ratio [INR], fibrinogen level, and red and white blood cell counts), and antibiotic administration (cefoperazone/sulbactam, ceftazidime, cefuroxime, ceftriaxone sodium, meropenem, and vancomycin). Age, sex, height, weight and BMI were recorded in the first patient visit. Other clinical and biochemical variables were collected longitudinally.

### Sample collection and SNP detection

At transplantation, ∼0.5 g of donor liver tissue was collected from the anterior margin of the right lobe during the routine zero-time biopsy. The use of these tissue samples was approved by the Ethics Committee of Shanxi Provincial People’s Hospital and Ethics Committee of First Hospital of Shanxi Medical University. Samples were placed in 1 mL RNAlater within 1.5 mL tubes, kept at 0–4 °C overnight, then stored at −80 °C. Before surgery, 3 mL of recipient peripheral blood was collected and preserved at −80 °C. Samples were outsourced to BGI Genomics Co., Ltd. (Shenzhen, China) for SNP analysis using the Sanger sequencing method. Variables, that is SNPs and clinical and biochemical data, detected in the study are shown in Supplementary Dataset 2.

### Data processing for non-compartmental PK parameters

Outliers were removed before any analyses using boxplot.stats() function in R. Missing tacrolimus trough concentrations were imputed by fitting a mixed-effects model to the log-transformed observed trough data and using model-predicted values to fill missing time points with the nlme package in R [26]. Differences between original and imputed pharmacokinetic parameters were evaluated using the two-sided paired Wilcoxon signed-rank test. Because intensive post-dose sampling was not available, pharmacokinetic analyses were based on longitudinal trough concentration-time profiles. Non-compartmental indices were calculated using the linear trapezoidal rule to estimate the area under the trough concentration-time curve over the monitoring window (AUC(C0,time)) [27]. This AUC represents a partial cumulative exposure during the early post-transplant phase rather than a conventional 0–24 h AUC for a dosing interval. In this cohort, 11 of 20 participants exhibited stabilization (plateau) of trough concentrations during follow-up. The maximum trough concentration (C0,max) was defined as the highest observed trough level for each participant, and the time to maximum trough concentration (t(C0,max)) was defined as the day on which this value occurred. These metrics were used as surrogate indicators of tacrolimus accumulation and time to steady-state attainment, rather than true single-dose peak concentration. Accumulation behavior was further characterized using an accumulation index, defined as the ratio of trough concentration at each time point to the first measurable trough concentration within the same subject, reflecting relative trough accumulation over time. Average accumulation ratio indicates the mean of the within-subject ratios. An alternative index for measuring the accumulation behavior was AUC(C0,time)-total dose ratio, defined as the ratio of AUC(C0,time) to total dose.

### Data processing for clinical measurements

For each participant, longitudinal clinical measurements, for example ALT, AST, TT, collected throughout the monitoring period were summarized to capture both the average physiological state and the baseline values at treatment initiation, with baseline variables annotated using the suffix ‘_baseline’. To further quantify the rate of change of each clinical laboratory variable over time at the individual level, nonlinear slopes were estimated using generalized additive models. For each participant and each clinical factor, a smooth trajectory was fitted using the mgcv::gam function in R with a cubic regression spline basis and restricted maximum likelihood estimation. The fitted smooth curve was then evaluated on a dense grid of 200 evenly spaced time points spanning the observed time range, and the instantaneous slope was approximated by numerical differentiation of consecutive predicted values. A single summary slope per participant per variable was derived as the mean derivative across time, representing the overall average rate of change of the laboratory value during the follow-up period. Variables for the rate of changes were annotated using the suffix ‘_trajectory’.

### Statistical analysis of correlations between variables and the PK of tacrolimus

Outliers in numeric variables were removed before any association analyses using boxplot.stats() function in R. To account for potential confounding by total dose, a dose-adjusted analysis was implemented using a residual approach in R. Briefly, both the covariate and the PK outcome were converted to ranks using rank function. For each ranked variable, a linear regression model with total dose as the independent variable was fitted using lm function. The model was specified as rank(outcome) = α_0_ + α_1_ × total dose + η, where rank(outcome) represents the ordered position of the PK metric. α_0_ is the intercept, and α_1_ quantifies the influence of dose on the outcome ranking. η denotes the residual component, reflecting the part of the outcome that cannot be attributed to dose. These residuals were extracted and used to represent the dose-independent variation in subsequent analyses. Spearman correlations between the two sets of residuals were then calculated using cor.test function to get significance levels. Results were retained only when at least 16 (80%) complete paired observations were available. For categorical covariates, dose-adjusted comparisons depended on outcome distribution. Normal distribution of variables was determined by shapiro.test function in R. For normal distributed outcomes (i.e. C0,max, t(C0,max), and AUC(C0,time)-total dose ratio, see details in Supplementary Dataset 3), the formula lm(outcome ∼ factor + total dose) was fitted and group P-values were obtained from anova or summary function. For non-normal outcomes, the variable was ranked using rank(), followed by fitting with lm(rank_outcome ∼ factor + total dose), with the factor P-value extracted from anova function. Associations with AUC(C0,time)-total dose ratio or AUC(C0,time) were not adjusted by total dose, consistent with its dose-normalized definition.

### Interaction and combined effect between rs2032582 genotype and cefoperazone/sulbactam administration on t(C0,max) of tacrolimus

A linear regression model was fitted with t(C0,max) as the dependent variable and recipient rs2032582 genotype, cefoperazone/sulbactam administration, and their interaction term as independent variables using the lm function in R. Alternatively, a composite variable integrating both factors was generated. The combined influence of genotype and antibiotic treatment on t(C0,max) was evaluated by dose-adjusted one-way ANOVA as described above.

### Prediction of protein structure and molecular docking

Three‐dimensional structural models of ABCB1 representing each genotype of the SNP rs2032582 were generated using ColabFold, which enables prediction by combining MMseqs2 homology search with AlphaFold2 algorithms [28]. Using these predicted protein models, molecular docking simulations were conducted *via* CB‑Dock2, a structure-based blind docking tool integrating a curvature-based cavity detection algorithm and template-based docking modules, to identify potential ligand binding sites and to compute binding affinities (Vina scores) for tacrolimus, cefoperazone, and sulbactam across the different genotypes [29]. The three‐dimensional structures of the small-molecule drugs were retrieved from the PubChem Compound database (NCBI). The top five high-affinity binding cavities detected by CB-Dock2 were listed. Each ligand was docked into the identified cavities using the AutoDock Vina algorithm, and for each docking pose, the contact residues, docking center coordinates, and cavity volumes were displayed for comparison across genotypes and ligands.

### Pharmacokinetic modeling of tacrolimus

All categorical variables were converted to numeric factors using integer encoding, standardized using the scale function, and missing values were imputed using the predictive mean matching algorithm by the mice function in R. Model development was implemented using the caret framework with repeated 3-fold cross-validation (20 repeats) in R. Multiple algorithms were tested, including linear regression (LM), random forest (RF), Bayesian generalized linear model (BGLM), boosted generalized linear model (GLM), support vector machine (SVM), k-nearest neighbor (KNN), extreme gradient boosting (xgbLinear, xgbTree), conditional inference forest (CFOREST), and Rborist. Variable importance was extracted from each model using the varImp function, and model performance was summarized by root mean square error (RMSE), R^2^, and mean absolute error (MAE) metrics. The best-performing models for each PK endpoint were selected based on the lowest RMSE. Model generalizability was further assessed by leave-one-out cross-validation (LOOCV). Predicted versus observed values were linearly regressed to estimate correlation coefficients (R) and P-values, and visualization was performed with shaded 95% confidence intervals.

## Results

### PK of tacrolimus

**Figure 1.**
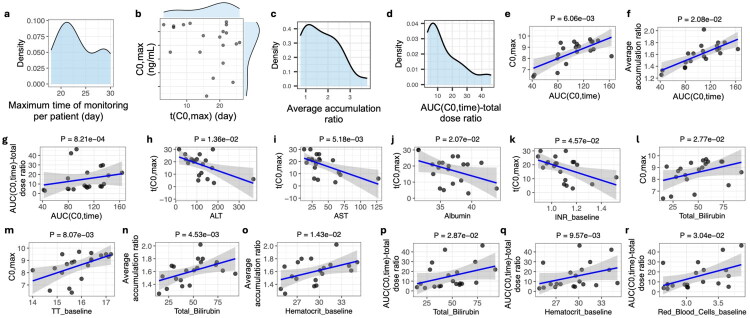
Associations between PK indices derived from longitudinal trough concentrations and clinical laboratory variables. Clinical laboratory variables represent average values during the monitoring period unless annotated with the suffix ‘_baseline’, which indicates measurements at treatment initiation. Full variable definitions and units are provided in Supplementary Dataset 2. (a–d) Distribution of key PK descriptors, including (a) maximum monitoring duration per participant, (b) the joint distribution of t(C0,max) and C0,max, (c) the average accumulation ratio, and (d) AUC(C0,time)-total dose ratio. (e–g) Correlations between cumulative exposure quantified by AUC(C0,time) and (e) C0,max, (f) average accumulation ratio, and (g) the AUC(C0,time)-total dose ratio. (h–k) Correlations between t(C0,max) and biochemical markers, including (h) ALT, (i) AST, (j) albumin, and (k) baseline INR. (l–m) Correlations between C0,max and (l) total bilirubin and (m) baseline TT. (n–o) Correlations between average accumulation ratio and (n) total bilirubin and (o) baseline hematocrit. (p–r) Correlations between the AUC(C0,time)-total dose ratio and (p) total bilirubin, (q) baseline hematocrit, and (r) baseline red blood cell count. For continuous covariates, dose-adjusted associations were evaluated using a residual approach in which both variables were rank-transformed, regressed on total dose, and correlations between residuals were assessed using Spearman’s method (see Methods). Detailed statistical results are provided in Supplementary Dataset 4.

Our cohort included 20 liver transplant recipients, with disease prior to transplantation summarized in Supplementary Dataset 1. Notably, none of the patients required vasopressor support or mechanical ventilation, and no patients were admitted to the intensive care unit prior to transplantation (Table 1). Monitoring durations ranged from 18 to 30 days and peaked at approximately 21 days (i.e. from the initiation of tacrolimus therapy to the last recorded concentration) (Figure 1a). Intermittent gaps in the longitudinal trough concentration profiles were filled to obtain one concentration per day using a mixed-effects model, yielding pharmacokinetic metrics comparable to those derived from the original data (Figure S1). Details of daily tacrolimus concentrations for each patient and the relationship between individual tacrolimus concentrations and cumulative dose are presented in Figure S2a and S^2^b, respectively.

**Table 1.** Demographic, clinical, and biochemical characteristics of participants in the cohort.

Characteristic	*N* = 20*
Sex	
Female	6 (30%)
Male	14 (70%)
Vancomycin	
No	14 (70%)
Yes	6 (30%)
Cefoperazone sulbactam	
No	4 (20%)
Yes	16 (80%)
Ceftriaxone sodium	
No	18 (90%)
Yes	2 (10%)
Meropenem	
No	12 (60%)
Yes	8 (40%)
Cefotaxime sodium	
No	19 (95%)
Yes	1 (5%)
Ceftazidime	
No	17 (85%)
Yes	3 (15%)
Cefuroxime	
No	18 (90%)
Yes	2 (10%)
AUC(C0,time)	105 (32)
Age (years)	48 (8)
Height (cm)	168 (8)
Weight (kg)	65 (11)
BMI	22.9 (3.5)
Hematocrit (%)	31.5 (12.7)
Serum creatinine (μmol/L)	66 (25)
Albumin (g/L)	36.46 (3.03)
Total bilirubin (μmol/L)	52 (25)
ALT (U/L)	163 (91)
AST (U/L)	67 (58)
APTT (s)	31.54 (3.11)
PT (s)	14.78 (1.59)
Fibrinogen (g/L)	2.82 (0.73)
INR	1.13 (0.12)
TT (s)	15.33 (0.79)
White blood cells (×10^9^/L)	8.1 (4.2)
Red blood cells (×10^12^/L)	3.4 (1.26)
Platelets (×10^9^/L)	146 (90)
Hematocrit_baseline (%)	29.21 (2.93)
Serum Creatinine_baseline (μmol/L)	73 (38)
Albumin_baseline (g/L)	35.04 (3.26)
Total bilirubin_baseline (μmol/L)	84 (61)
ALT_baseline (U/L)	410 (326)
AST_baseline (U/L)	190 (279)
APTT_baseline (s)	32 (7)
PT_baseline (s)	13.71 (4.12)
Fibrinogen_baseline (g/L)	2.53 (0.83)
INR_baseline	1.17 (0.37)
TT_baseline (s)	16.14 (1.18)
White blood cells_baseline (×10^9^/L)	7.1 (5.5)
Red blood cells_baseline (×10^12^/L)	3.14 (0.39)
Platelets_baseline (×10^12^/L)	55 (43)

Note: Full names and units of the variables are shown in supplementary Dataset 1. Biochemical factors are average values throughout the monitoring period and baseline measurements are annotated with the suffix ‘_baseline’. *n (%); Mean (SD).

Tacrolimus trough-based exposure dynamics were characterized using the t(C0,max), C0,max, the average accumulation ratio, and AUC(C0,time)-total dose ratio, which together describe the dynamics of trough concentration build-up and stabilization over time rather than classical single-dose absorption kinetics. The time to maximum observed trough concentration, t(C0,max), rather than absorption-phase t(C0,max), varied widely from 3 to 26 days, with a median of 19 days (Figure 1b). C0,max ranged from approximately 5–9 ng/mL, and there was no significant linear correlation between C0,max and t(C0,max), indicating that patients who reached peak trough concentrations earlier did not necessarily exhibit lower plateau trough (C0,max) values compared to those with later t(C0,max).

The average accumulation ratio, reflecting relative trough accumulation over time within individuals, exhibited a right‐skewed distribution, suggesting that a small subset experienced markedly higher accumulation rates (Figure 1c). Additionally, AUC(C0,time)-total dose ratio, which also reflects relative cumulative trough exposure over the observation period, showed a similar trend to that of the average accumulation ratio (Figure 1d).

### Clinical laboratory factors associated with the PK of tacrolimus

Not surprisingly, AUC(C0,time) was positively correlated with C0,max, indicating that individuals with greater cumulative exposure tended to reach higher peak trough levels during follow-up (Figure 1e). Additionally, AUC(C0,time) was positively correlated with the average accumulation ratio (Figure 1f) and AUC(C0,time)-total dose ratio (Figure 1g), suggesting that greater cumulative exposure was accompanied by more pronounced trough accumulation over time. However, t(C0,max) was not significantly associated with AUC(C0,time), suggesting that the timing of peak trough concentration is at least not solely determined by cumulative exposure (row 15 in Supplementary dataset 4).

Unless otherwise specified, clinical laboratory factors represent the average values measured over the monitoring period. ALT and AST, two biomarkers of liver injury, together with serum albumin and baseline INR, markers of hepatic synthetic and coagulation functions, were significantly negatively correlated with t(C0,max) (Figure 1h–k). Meanwhile, C0,max was significantly correlated with total bilirubin (Figure 1l) and baseline TT (Figure 1m), two clinical biomarkers for liver function and coagulation, respectively.

The average accumulation ratio had positive correlations with total bilirubin, and baseline level of hematocrit (Figure 1n–o). Similarly, total bilirubin and baseline thrombin time were positively correlated with AUC(C0,time)-total dose ratio, an alternative index for relative trough accumulation (Figure 1p–q). Additionally, red blood cells at baseline were also positively correlated with AUC(C0,time)-total dose ratio (Figure 1r).

### Genotype- and drug-related associations with tacrolimus pharmacokinetics

As introduced above, *ABCB1* and *CYP3A* play essential roles in the PK of tacrolimus. Accordingly, SNPs in these two gene groups were examined, including rs1045642 and rs2032582 for *ABCB1* in recipients, as well as rs776746 (CYP3A5*3), rs15524 (*CYP3A5*), rs2242480, rs2246709, and rs4646437 (*CYP3A4*) for *CYP3A* genes in donors.

**Figure 2.**
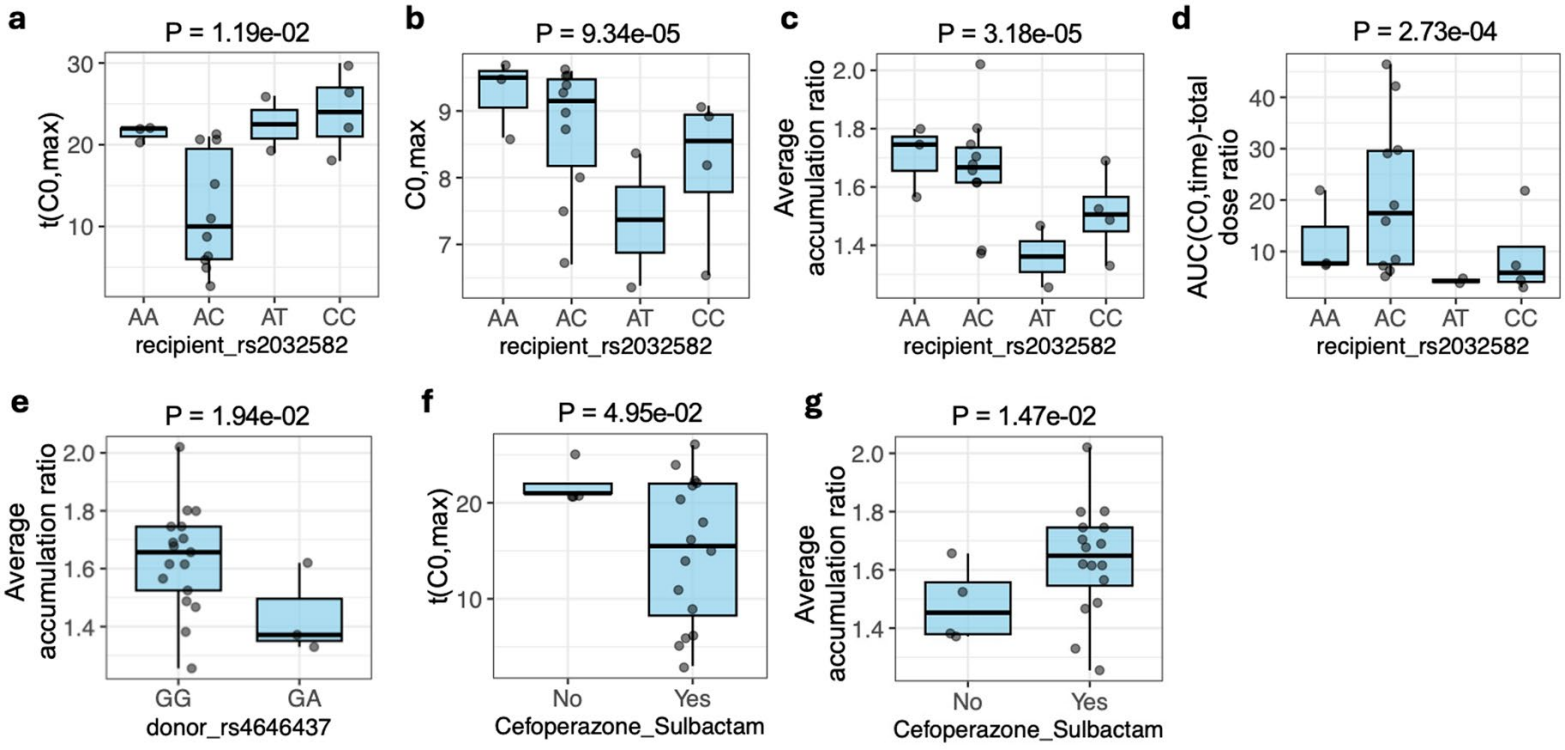
Genetic and antibiotic treatment factors associated with tacrolimus trough–derived PK indices. (a–d) Associations between recipient rs2032582 genotype and (a) t(C0,max), (b) C0,max, (c) average accumulation ratio, and (d) the AUC(C0,time)-total dose ratio. (e) Association between donor rs4646437 genotype and average accumulation ratio. (f–g) Comparisons according to cefoperazone/sulbactam exposure for (f) t(C0,max) and (g) average accumulation ratio. For categorical covariates, comparisons were performed using linear regression models including total dose as a covariate; normally distributed outcomes were analyzed on the original scale, whereas non-normal outcomes were rank-transformed based on results from the Shapiro-Wilk test. P values were derived from the corresponding model terms (see Methods). Detailed statistical results are provided in Supplementary Dataset 5.

The variant rs2032582 results in a non-synonymous amino-acid substitution at position 893 in the encoded P-gp transporter [30]. In genotype analyses, rs2032582 was significantly associated with t(C0,max), with carriers of the AC genotype exhibiting a shorter t(C0,max) compared with other genotypes (Figure 2a; Supplementary dataset 5, row 13). In addition, C0,max differed significantly across rs2032582 genotypes (Figure 2b), with the AT genotype showing the lowest C0,max. Notably, although the AC genotype was associated with a shorter t(C0,max), its corresponding C0,max remained relatively high, indicating that AC carriers reached higher trough concentrations earlier during the observation period. Consistent with the results, AC carriers had higher levels of drug accumulation as quantified by average accumulation ratio and AUC(C0,time)-total dose ratio (Figure 2c and d). Although recipients with the rs2032582 AA genotype exhibited a longer t(C0,max) than AC carriers (Figure 2a), their average accumulation ratio was comparable, whereas the AUC(C0,time)-total dose ratio differed between genotypes (Figure 2d). This pattern is interpreted in detail in the Discussion.

The average tacrolimus accumulation ratio was significantly associated with rs4646437 (a SNP in the CYP3A4 gene) in donors, with the GG genotype exhibiting higher accumulation-ratio levels compared to the GA genotype (Figure 2e). The observation is consistent with prior studies demonstrating that the CYP3A4 rs4646437 variant is associated with altered tacrolimus dose-adjusted concentrations [31].

As described in the Methods, antibiotics with known drug-drug interactions with tacrolimus (e.g. rifampin, phenytoin/carbamazepine, and clarithromycin) were excluded from the study. The effects of the remaining antibiotics, i.e. cefoperazone/sulbactam, ceftazidime, ceftriaxone sodium, cefuroxime, meropenem, and vancomycin, on tacrolimus PK were subsequently evaluated. Patients receiving cefoperazone/sulbactam exhibited a shorter t(C0,max) and a higher average accumulation ratio than those not receiving cefoperazone/sulbactam (Figure 2f and g). Therefore, cefoperazone/sulbactam was associated with accelerated tacrolimus trough accumulation during the early post-transplant period.

### Potential mechanisms underlying the effect of recipient rs2032582 on tacrolimus t(C0,max)

**Figure 3.**
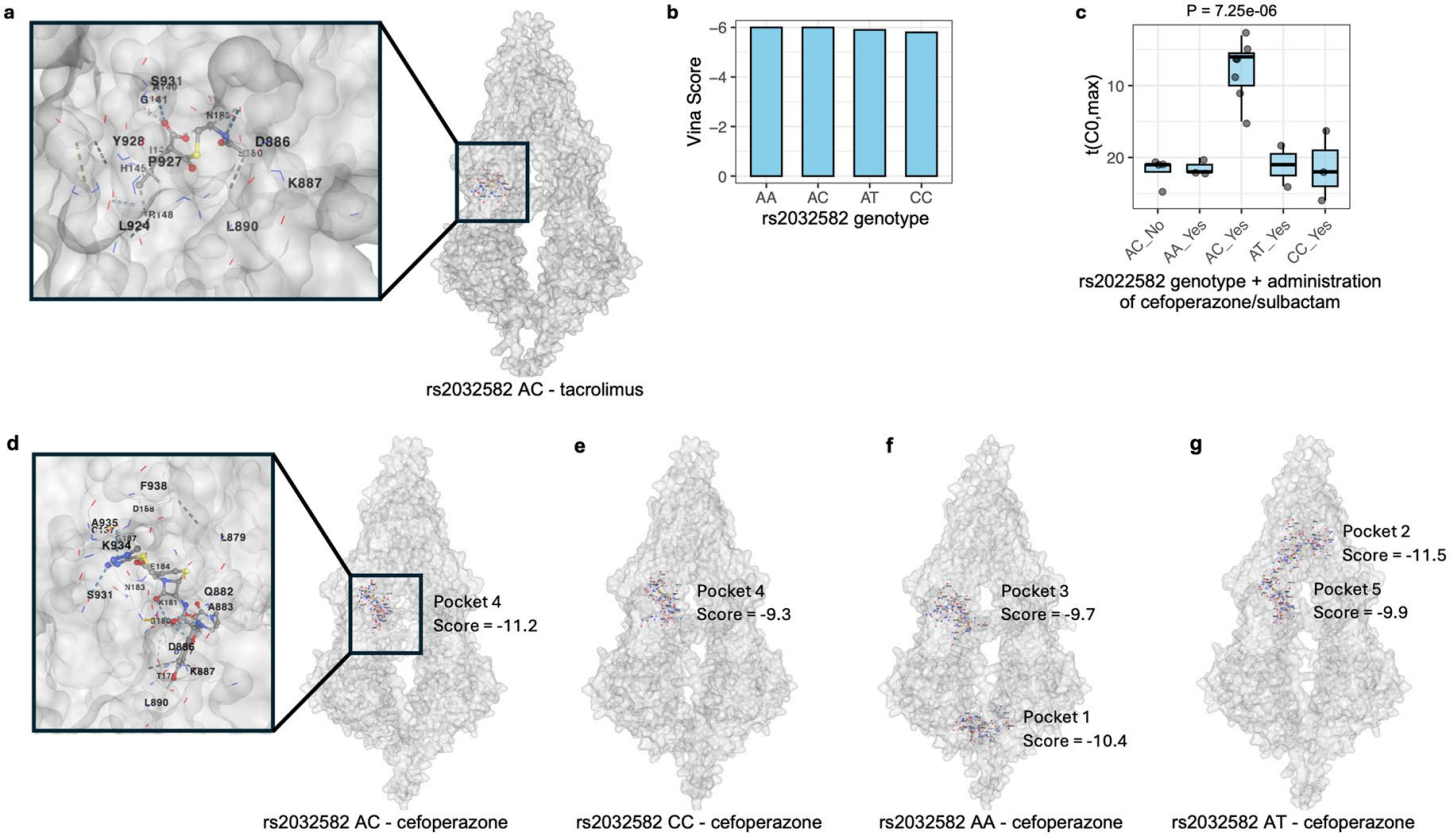
The structural basis by which ABCB1 rs2032582 variants may influence absorption kinetics of tacrolimus. Predicted protein structures for each ABCB1 rs2032582 genotype were obtained using ColabFold, and molecular docking simulations were conducted with CB-Dock2 to estimate binding affinities between drugs and ABCB1 variants. (a) The best docking conformation of tacrolimus with the ABCB1 rs2032582 AC variant is shown, highlighting key amino-acid residues involved in ligand-protein interactions. The full set of docking conformations for other genotypes is illustrated in Figure S3 and Supplementary dataset 6. (b) The resulting Vina scores between tacrolimus and rs2032582 genotypes are presented to compare relative docking energies. (c) The combined effect between rs2032582 genotype and concomitant administration of cefoperazone/sulbactam on the t(C0,max) of tacrolimus analyzed using a one-way ANOVA is shown. (d–g) Docking analyses between cefoperazone and ABCB1 rs2032582 variants (AC, CC, AA, and AT) are shown with details shown in Figure S5 and Supplementary dataset 7. When the best docking center overlaps with the ABCB1-tacrolimus binding site, that pocket and its Vina score are displayed; otherwise, both the overlapping pocket and the best docking pocket are annotated.

Three-dimensional structures of ABCB1 carrying different rs2032582 genotypes were predicted by ColabFold. Based on the predicted structures, docking analyses using CB-Dock2 indicated that rs2032582 is positioned within the central pocket for the binding of ABCB1-tacrolimus, in proximity to key binding residues, e.g. Leu890, Lys887, and Asp886, suggesting that this variant could influence the local conformation of the tacrolimus-binding region (Figure 3a). However, ABCB1 exhibited relatively weak binding with tacrolimus, with the Vina docking scores averaging around −6 across all rs2032582 genotypes (Figures 3b, S^3^, and column I in Supplementary dataset 6). Additionally, best docking sites were highly consistent among the ABCB1 variants, indicating that the rs2032582 polymorphism did not substantially alter the overall binding strength or pocket location of tacrolimus within ABCB1.

Since the administration of cefoperazone/sulbactam was also found to be associated with the t(C0,max) of tacrolimus (Figure 2f), the interaction between rs2032582 genotype and cefoperazone/sulbactam administration on t(C0,max) (Fig. S4a) was evaluated using a linear regression model (Figure S4b). The model indicated that approximately 77% of the variation (adjusted R^2^) in t(C0,max) was accounted for by the two predictors. However, because all patients without cefoperazone/sulbactam administration carried the rs2032582 AC genotype, the two factors were collinear and thus the interaction could not be statistically analyzed. Therefore, the two variables were merged into a combined genotype-treatment factor, as shown in Figure 3c, and its association with t(C0,max) was quantified using one-way ANOVA adjusted by total dose. The analysis indicated a significant combined effect of rs2032582 genotype and cefoperazone/sulbactam administration on t(C0,max), with patients carrying the AC genotype and receiving cefoperazone/sulbactam exhibiting an earlier t(C0,max) (Figure 3c).

Based on the observed combined effect shown above and evidence that ABCB1 polymorphism influences the PK of tacrolimus [32,33]. It was hypothesized that cefoperazone or sulbactam may compete with tacrolimus for binding to ABCB1, thereby inhibiting the ABCB1-mediated efflux of tacrolimus into the gastrointestinal lumen; this competitive mechanism could enhance tacrolimus absorption and may be further modulated by rs2032582. To test this hypothesis, docking analyses were conducted between ABCB1 variants and cefoperazone. These findings may be consistent with the proposed competitive binding mechanism, as cefoperazone exhibited the strongest binding affinity toward the AC genotype (Figure 3d–g, S^5^, and column I in Supplementary dataset 7), with a markedly higher affinity than that observed between ABCB1 and tacrolimus. Moreover, the cefoperazone binding site overlapped with that of ABCB1-tacrolimus, with substantial overlap in key interacting residues between the two complexes located near position 893, corresponding to the rs2032582 variant (Figure 3a and d). In contrast, for the CC genotype, cefoperazone was also bound at an overlapping site but with lower affinity (Figures 3e and S^5^), whereas the AA and AT genotypes displayed distinct preferred docking pockets that differed from those of tacrolimus (Figures 3f, g, and S5). Compared with cefoperazone, the interaction between sulbactam and ABCB1 was weaker, suggesting that sulbactam exerts a lesser influence on the t(C0,max) of tacrolimus when cefoperazone and sulbactam were co-administered (column I of Supplementary dataset 8).

### Pharmacokinetic modeling of tacrolimus

**Figure 4.**
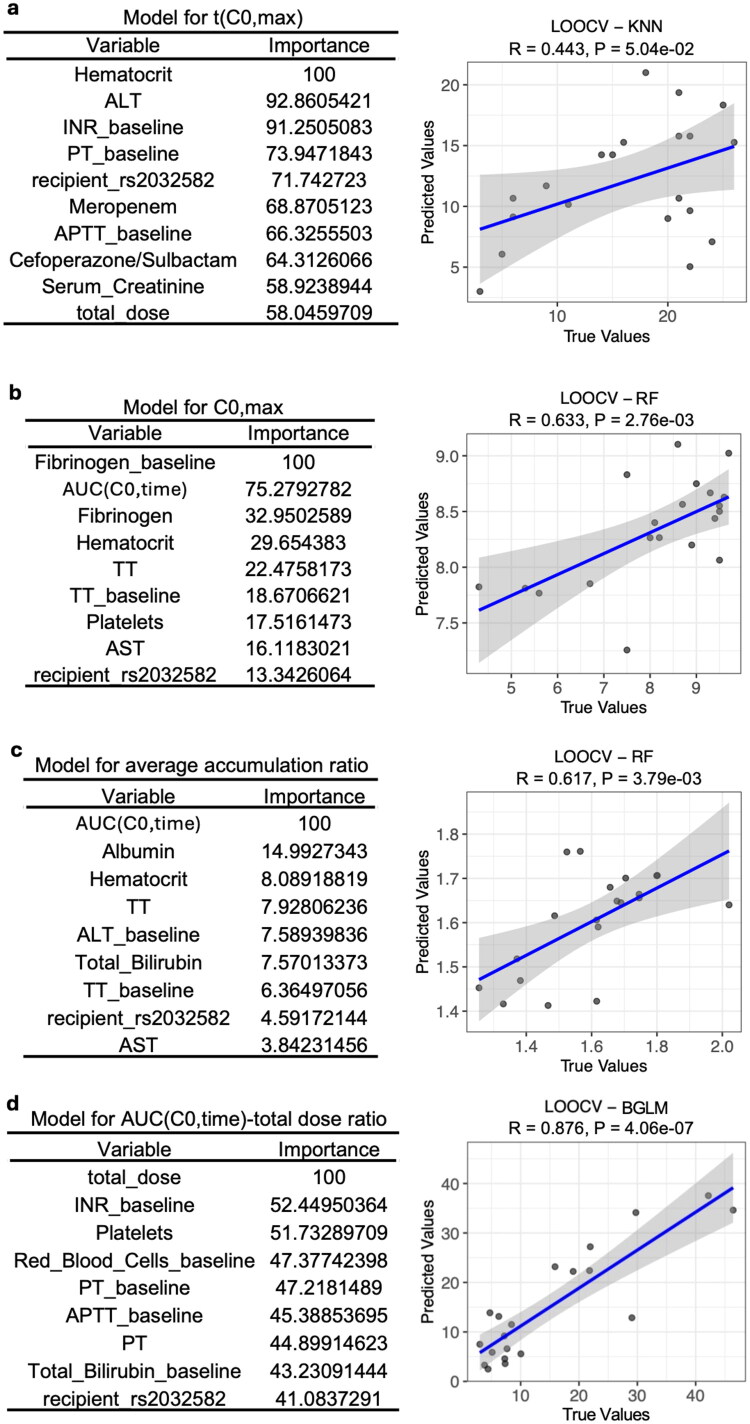
Pharmacokinetic modeling of tacrolimus. Machine-learning models were trained to identify factors associated with (a) t(C0,max), (b) C0,max, (c) average accumulation ratio, and (d) AUC(C0,time)-total dose ratio of tacrolimus using k-nearest neighbors (KNN), random forest (RF), RF, and Bayesian generalized linear model (BGLM), respectively. The left panels list the top predictive variables ranked by relative importance in the best-performing model. The right panels show correlations between model-predicted and observed values obtained by leave-one-out cross-validation (LOOCV), with performance evaluated by Pearson correlation. R- and *p*-values indicate correlation coefficient and the corresponding significance.

Machine-learning modeling identified distinct predictors for different trough-based PK surrogate parameters of tacrolimus. For t(C0,max), the KNN algorithm proved optimal, as indicated by the lowest RMSE (column B in Supplementary dataset 9), and important variables in the KNN model included hematocrit, ALT, baseline INR, baseline PT, recipient rs2032582 genotype, meropenem use, and cefoperazone/sulbactam administration (the first panel from left in Supplementary dataset 10). A LOOCV modeling strategy yielded a moderate correlation coefficient of 0.443 between predicted and observed t(C0,max) (Figure 4a). The significant role of hematocrit aligns with previous evidence that hematocrit markedly affects tacrolimus disposition and whole-blood concentration measurements [34]. For C0,max, a RF model highlighted baseline fibrinogen, AUC(C0,time) (quantifying systemic exposure), hematocrit, baseline TT, platelets, AST, and recipient rs2032582 genotype as the most influential factors, achieving a correlation coefficient of 0.633 between predicted and observed C0,max (Figure 4b and the second panel in Supplementary dataset 10). Regarding the average accumulation ratio, the RF model identified AUC(C0,time), albumin, hematocrit, and the recipient rs2032582 genotype among the top ten predictors, achieving moderate predictive performance (*R* = 0.617; Figure 4c and the third panel of Supplementary Dataset 10). Finally, a Bayesian generalized linear model (BGLM) was constructed to predict the AUC(C0,time)-to-total dose ratio, in which total dose, baseline INR, platelet count, recipient rs2032582 genotype, and additional variables ranked among the top ten predictors (Figure 4d and the fourth panel of Supplementary Dataset 10). The *R* value was 0.876, indicating strong predictive performance.

## Discussion

This study systematically characterized the early PK of tacrolimus in liver transplant recipients and uncovered clinical, biochemical, and genetic factors associated with interindividual variability in tacrolimus trough exposure dynamics, suggesting the multifactorial nature of the PK of tacrolimus variability. The findings reveal that both hepatic function-related biomarkers and the gene-drug combined effect significantly modulate the PK of tacrolimus in the first postoperative month. However, these findings should be interpreted as potential modulators of trough accumulation kinetics rather than direct determinants of absorption rate or instantaneous bioavailability.

A central discovery of this study is the combined effect between the *ABCB1* rs2032582 genotype and cefoperazone/sulbactam administration on tacrolimus t(C0,max). One plausible mechanistic explanation is that cefoperazone may interfere with ABCB1 (P-gp)-mediated intestinal efflux of tacrolimus in a genotype-dependent manner, thereby altering exposure dynamics. Molecular docking analyses suggested that cefoperazone may bind within a region of ABCB1 overlapping or adjacent to the tacrolimus binding site near residue 893, raising the possibility of competitive inhibition of P-gp-mediated transport. If operative, such an interaction could transiently increase intestinal tacrolimus uptake and shift the timing of peak exposure across different rs2032582 genotypes. Importantly, this interpretation remains hypothesis-generating and computational in nature. While it is consistent with prior *in vitro* evidence that tacrolimus is an ABCB1 substrate [35] and that pharmacologic inhibition of P-gp can increase tacrolimus bioavailability [36], the present study does not provide direct experimental confirmation of transporter-level competition. Definitive validation will require *in vitro* transport assays (e.g. ABCB1-overexpressing cell systems) as well as clinical pharmacodynamic or drug-drug interaction studies specifically designed to test cefoperazone-mediated modulation of tacrolimus transport.

Some previous studies have reported significant associations between ABCB1 variants and tacrolimus blood concentrations or dose requirements [32,37,38]; in contrast, other studies have found no significant impact of ABCB1 polymorphisms on tacrolimus pharmacokinetic parameters [39,40]. These discrepant findings may reflect differences in ethnic background, concomitant medications, and clinical settings, as well as the influence of additional covariates such as hepatic function. Systematic reviews and meta-analyses similarly highlight substantial heterogeneity across studies and emphasize caution in the clinical interpretation of ABCB1 genotypes for tacrolimus dosing decisions [41]. Taken together, our findings add to this complex and heterogeneous literature and underscore the need for more detailed studies incorporating a broader and more comprehensive set of genetic, pharmacokinetic, and clinical variables.

From a clinical perspective, the observed combined effect suggests that certain patients, particularly rs2032582 AC carriers, may experience altered early tacrolimus exposure dynamics during antibiotic co-administration. Although these findings are not aimed to justify routine preemptive dose adjustment, they support the rationale for closer early therapeutic drug monitoring and heightened clinical vigilance during cefoperazone exposure in genetically susceptible individuals. Genotype information, when available, may therefore aid in risk stratification and inform the intensity of early monitoring rather than dictate fixed dosing decisions. Prospective studies are required to determine whether genotype-guided monitoring or adaptive dosing strategies improve early post-transplant outcomes.

Despite a longer t(C0,max) in rs2032582 AA carriers than in AC carriers, the average accumulation ratios were similar, whereas the AUC(C0,time) normalized by total dose differed. This is not contradictory because the metrics capture different aspects of trough behavior. The accumulation ratio reflects a relative fold change from the initial concentration and is therefore dependent on baseline levels. In contrast, the AUC(C0,time)-total dose ratio integrates concentrations across the entire monitoring period and represents the absolute exposure achieved per unit dose, largely independent of the starting value. Consequently, AA carriers may accumulate more slowly and reach a comparable fold increase, yet still exhibit lower overall dose-normalized exposure. Consistent with this distinction, t(C0,max) was not correlated with AUC(C0,time), supporting the interpretation that the timing of peak trough concentration reflects physiological processes distinct from cumulative exposure. The divergent associations of laboratory markers across these indices further imply that hepatic injury, inflammatory status, and coagulation function differentially influence the magnitude versus timing of tacrolimus accumulation.

Beyond transporter-mediated effects, our findings show that laboratory markers of hepatic function, coagulation, and inflammation are associated with tacrolimus trough accumulation early after transplantation. Higher ALT and AST together with lower albumin were linked to a shorter t(C0,max), whereas elevated white blood cell counts were associated with delayed t(C0,max), suggesting that hepatocellular injury and inflammatory status [42–45] influence the timing of accumulation rather than overall exposure. Total bilirubin and baseline TT [46,47], were positively associated with C0,max, the average accumulation ratio, and the AUC(C0,time)-total dose ratio, indicating greater and more sustained exposure in recipients with impaired hepatic processing or altered coagulation. Because tacrolimus undergoes extensive hepatic metabolism and biliary handling [8,48], reduced liver function may decrease presystemic clearance and increase dose-normalized concentrations. This mechanism is consistent with metabolism by CYP3A enzymes and transport *via* P-glycoprotein in hepatic and intestinal tissues [6,7].

The machine-learning analysis adds another layer by showing that the PK of tacrolimus in the early post-transplant setting is not driven by a single dominant pathway. Rather, as shown in Supplementary Dataset 10, although the specific variables identified as important differed across models, biomarkers reflecting hepatic function and hematologic status, and genetic factors consistently comprised the majority of the top ten predictors in each model. Some prior works generally treat antibiotics and coagulation function as secondary factors [49,50]. In contrast, our models elevate these variables to primary drivers, demonstrating that they explain additional pharmacokinetic variability beyond that captured by metabolic genotypes or standard hepatic function laboratory indices alone. For instance, antibiotics may directly influence transporter activity, while coagulation and other biomarkers, e.g. serum creatinine, serve as integrated indicators of the graft’s metabolic readiness rather than passive reflections of function (e.g. platelets served as an important factor in the modeling of C0,max and AUC(C0,time)-total dose ratio).

This study is limited by the relatively small sample size, which restricts statistical power and increases the risk of false-negative findings. The limited cohort size also constrains generalizability, particularly to populations with different racial or genetic backgrounds. In addition, the modest sample size increases the risk of overfitting and instability in machine-learning models, despite the use of cross-validation strategies. Furthermore, renal function was represented only using serum creatinine measurements, as standardized estimates of glomerular filtration rate and other renal indices were not consistently available during the immediate post-transplant period. The absence of these additional renal parameters may have limited the ability to fully capture renal-related contributors to variability in tacrolimus PK. Meanwhile, cold and warm ischemic times were not available in this cohort and therefore were not adjusted for. Because ischemic exposure can influence early graft injury and metabolic capacity, residual confounding related to donor quality cannot be excluded. Additionally, as this was an observational study, the relationships identified between clinical biomarkers and tacrolimus PK, including key predictors highlighted by the machine-learning models, should be interpreted as associations. Therefore, studies with larger sample sizes, together with more comprehensive laboratory profiling and direct measures of metabolic capacity, are needed to validate these findings and establish their robustness and clinical applicability.

Conceptually, this study separates early tacrolimus kinetics into two coordinated domains, ‘input control’ that governed by intestinal transport and antibiotic-modulated efflux (ABCB1/P-gp) and ‘handling capacity’ that governed by graft synthetic function and donor metabolism, and argues that safe, effective, individualized dosing in the first postoperative month will manage both simultaneously rather than only titrating to a trough target.

## Supplementary Material

Supplementary_figures.docx

SI Datasets 0211.xlsx

## Data Availability

All data involved in this study are available from the first author, Jinlin Guo (email: guojinlin@sxmu.edu.cn), upon reasonable request.
