# Supplementary material for: Genetic and clinical determinants of tacrolimus accumulation in early liver transplantation: evidence for a combined genotype-antibiotic effect
Source: Ann Med. 2026 Mar 12;58(1):2643032. doi: 10.1080/07853890.2026.2643032 (PMC12990272; doi:10.1080/07853890.2026.2643032)
Supplement: Supplementary_figures.docx [file IANN_A_2643032_SM7900.docx]

# Supplementary Figures

**Fig. S1 Comparison between original and imputed PK metrics.**


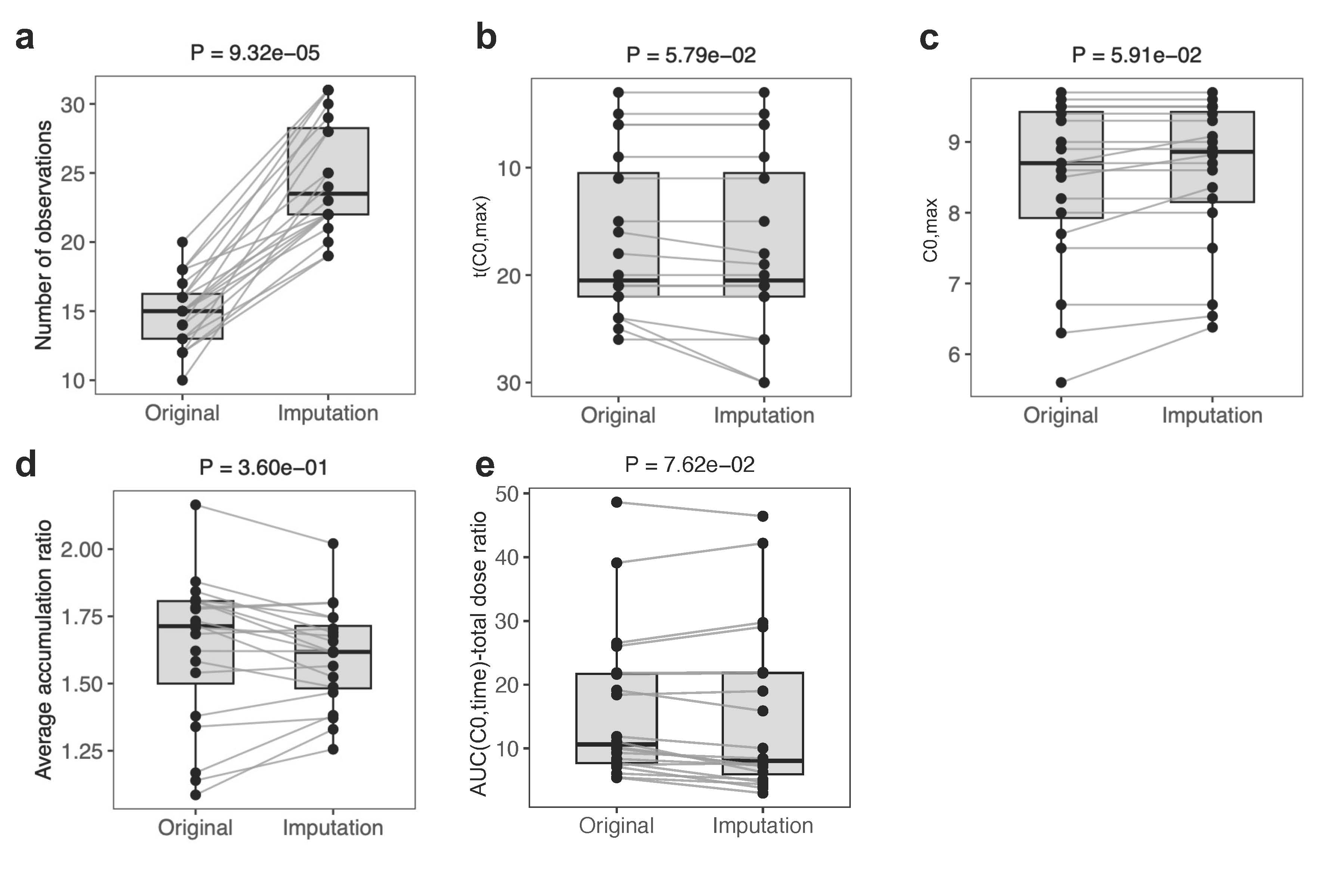


Intermittent gaps in the longitudinal trough concentration profiles were filled using a mixed-effects model. Difference for **(a)** number of observations, **(b)** t(C0,max), **(c)** C0,max, **(d)** the average accumulation ratio, and **(e)** AUC(C0,time)-total dose ratio were measured by two-sided paired Wilcoxon signed-rank test.

**Fig. S2 Concentration dynamics of tacrolimus and cumulative dose.**


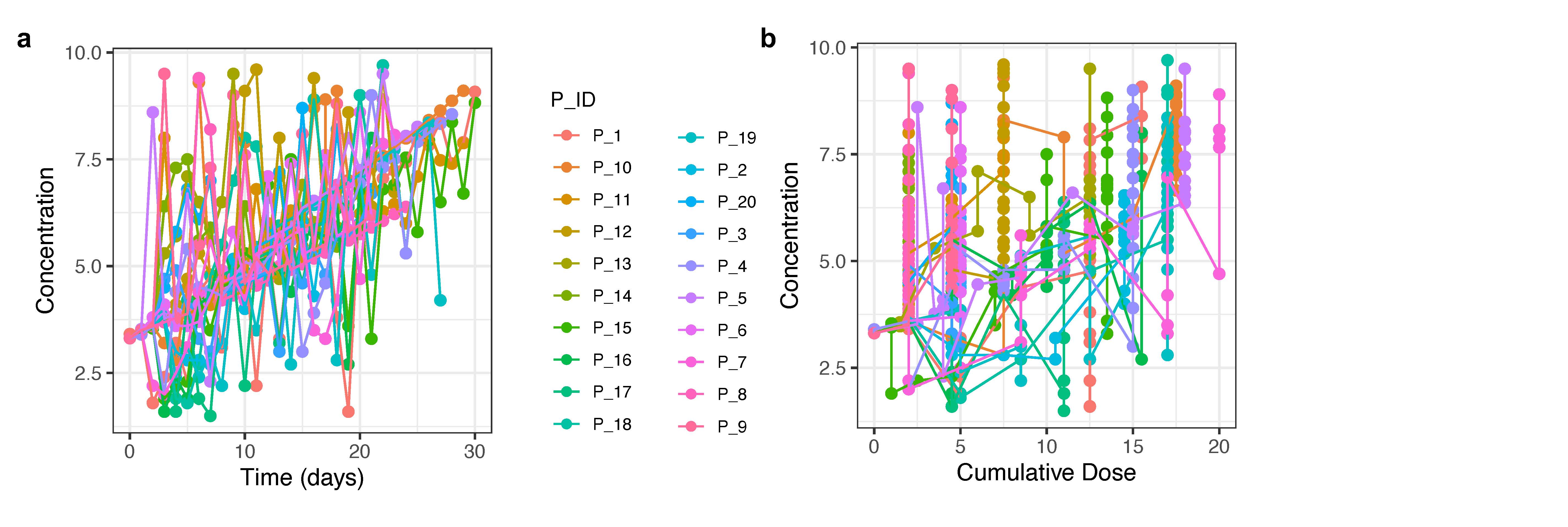


**(a)** Time-course profiles of tacrolimus blood concentration for each liver transplant recipient during the observation period, with individual trajectories color-coded by participant identifier (P_ID). **(b)** Relationship between tacrolimus concentration and cumulative administered dose, visualized across all participants to assess interindividual variability in concentration-dose proportionality.

**Fig. S3 The best docking conformation of tacrolimus with the ABCB1 rs2032582 variants.**


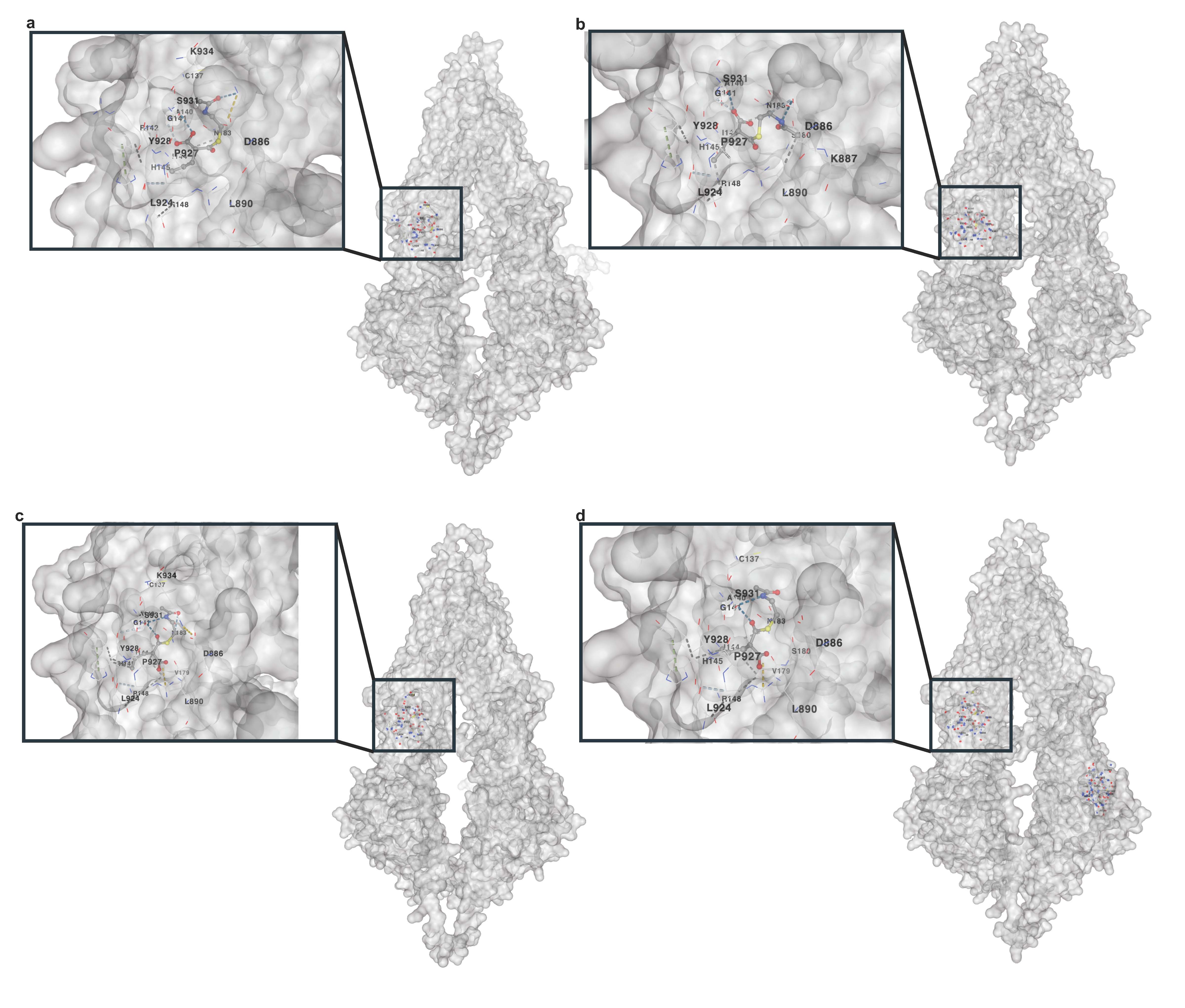


Docking analyses between cefoperazone and ABCB1 rs2032582 genotypes AA **(a)**, AC **(b)**, AT **(c)**, and CC **(d)** are shown.

**Fig. S4 Interaction analysis of recipient rs2032582 genotype and cefoperazone/sulbactam administration on tacrolimus AUC(C0,time).**


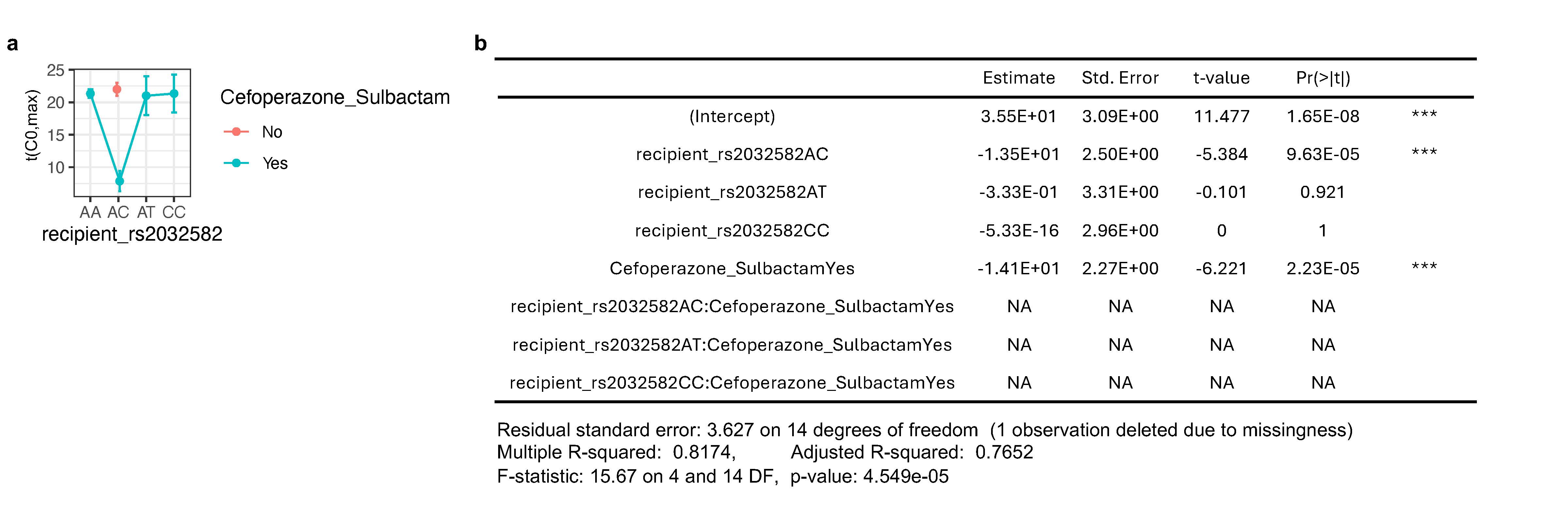


(a) Visualization of tacrolimus AUC(C0,time) across rs2032582 genotypes and cefoperazone/sulbactam administration groups. (b) Summary of the linear regression model results, with AUC(C0,time) as the dependent variable and rs2032582 genotype, cefoperazone/sulbactam administration, and their interaction term as independent variables. The model was fitted using the lm function in R. Model performance metrics (R^2^, adjusted R^2^, and F-statistic) are shown at the bottom.

**Fig. S5 Docking conformation of cefoperazone with the ABCB1 rs2032582 variants.**


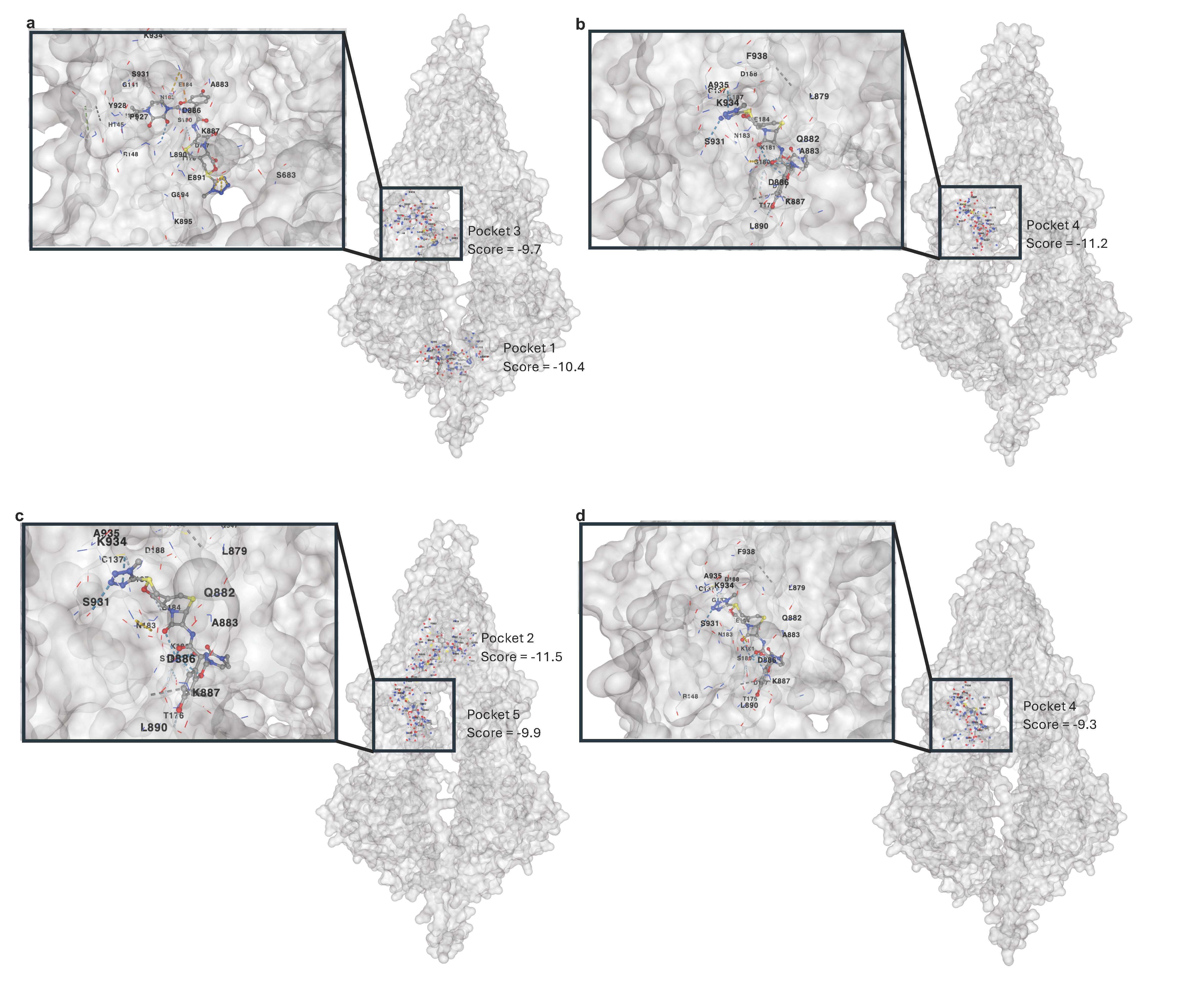


Docking analyses between cefoperazone and ABCB1 rs2032582 genotypes AA **(a)**, AC **(b)**, AT **(c)**, and CC **(d)** are shown.
